# Determining an appropriate fosfomycin (ZTI-01) dosing regimen in pneumonia patients by utilizing minimal PBPK modeling and target attainment analysis

**DOI:** 10.1128/aac.01869-24

**Published:** 2025-05-05

**Authors:** Jomana Al Hroot, Joshua Reeder, Xuanzhen Yuan, Kenan Gu, Emmanuel B. Walter, Lindsay Boole, Loretta G. Que, Guohua An

**Affiliations:** 1 Department of Pharmaceutical Sciences and Experimental Therapeutics, College of Pharmacy, University of Iowa 15509, Iowa City, Iowa, USA; 2 Division of Microbiology and Infectious Diseases, National Institute of Allergy and Infectious Diseases 540429, Bethesda, Maryland, USA; 3 Duke Human Vaccine Institute, Duke University School of Medicine 12277 , Durham, North Carolina, USA; 4 Department of Pediatrics, Duke University School of Medicine 12277 , Durham, North Carolina, USA; 5 Division of Pulmonary, Allergy, and Critical Care Medicine, Duke University of School of Medicine 12277 , Durham, North Carolina, USA; The Peter Doherty Institute for Infection and Immunity, Melbourne, Victoria, Australia

**Keywords:** m-PBPK, target attainment analysis, fosfomycin, pneumonia, repurposing

## Abstract

Fosfomycin, a broad-spectrum antibiotic used for uncomplicated cystitis, represents a potential promising candidate in combating resistant pneumonia. To facilitate the transition of fosfomycin to broader indications, including pneumonia, a minimal physiologically based pharmacokinetics (m-PBPK) model for fosfomycin was developed based on data from plasma, epithelial lining fluid (ELF), and alveolar macrophages (AMs) obtained from 37 healthy participants in a recently completed intrapulmonary PK study. Utilizing this mechanistic m-PBPK model enabled us to predict drug concentrations at the infection site in pneumonia patients, taking into consideration the pathophysiological changes occurring during the infection. Our prediction shows that the drug concentrations at the infection site reduced, while plasma levels remain unchanged. Monte Carlo simulations were conducted to evaluate the probability of target attainment (PTA) for various dosing regimens infused over 1 h against major hospital-acquired pneumonia pathogens in plasma and ELF. Our PTA analysis suggested that if plasma concentrations are the appropriate efficacy indicator, a dose of 4 g q8h is sufficient for *Pseudomonas aeruginosa and Staphylococcus aureus* infections. However, if ELF concentrations are a more accurate indicator, this dose is only effective for *S. aureus* pneumonia. For *P. aeruginosa* pneumonia, a dose of 6 g q8h is recommended, with an even higher dose of 8 g q8h necessary for pneumonia patients. In conclusion, our model provides critical insights into fosfomycin dosing for pneumonia treatment, guiding clinical study design. Furthermore, it serves as a platform to evaluate intrapulmonary pharmacokinetics for other antibiotics.

## INTRODUCTION

Hospital-acquired pneumonia (HAP) is one of the most common hospital-acquired infections, accounting for 22% of all reported cases (1). It is primarily caused by *Staphylococcus aureus*, *Pseudomonas aeruginosa*, and *Acinetobacter baumannii,* all of which belong to the ESKAPE pathogens (*Enterococcus faecium, Staphylococcus aureus, Klebsiella pneumoniae, Acinetobacter baumannii, Pseudomonas aeruginosa,* and *Enterobacter* species), recognized by the WHO as a priority concern due to their role in life-threatening and antimicrobial resistance infections (2, 3). Globally, HAP represents the leading cause of hospital infections in intensive care units (ICUs), with estimated mortality rates of 20%–30% (3).

For many decades, the standard treatment for pneumonia was beta-lactams, a class of antibiotics that has been ineffective in fighting against resistant bacteria. To address this issue, various strategies have been developed, including developing newer generations, such as cephalosporins and carbapenems, or combining beta-lactams with beta-lactamase inhibitors (3). Unfortunately, the pathogens that can cause HAP often produce extended-spectrum beta-lactamases (ESBLs) or have developed resistance mechanisms against carbapenems (4–6). With limited treatment options available for HAP, there has been a suggestion to re-evaluate and use some of the “forgotten” older antibiotics (7, 8).

One such candidate is fosfomycin, an old antibiotic first identified in 1969 and approved in 1996 for the treatment of uncomplicated cystitis. Fosfomycin is a bactericidal antibiotic that inhibits MurA (UDP-N-acetylglucosamine enolpyruvyl transferase), an enzyme catalyzing the first step of bacterial cell wall synthesis, thereby disrupting cell wall formation (9). It has been reported that fosfomycin is effective in treating various infections caused by resistant pathogens (8, 10, 11). When administered via IV infusion, fosfomycin rapidly penetrates into the human tissues, achieving therapeutically relevant concentrations in serum, urine, and various organs, including the lungs (12). Clinical data showed that fosfomycin is well-tolerated, undergoes renal excretion with an elimination half-life of 1.9 to 3.9 h, has negligible protein binding, and has a volume of distribution (Vd) of 9 to 30 L after IV administration (13). Its antimicrobial efficacy is concentration-dependent, meaning that higher drug concentrations lead to greater microbial killing, and its effectiveness is often associated with the area under the curve [AUC]/MIC ratios (14).

Based on its favorable efficacy, safety, and pharmacokinetics profiles, fosfomycin represents a potential promising candidate in combating resistant pneumonia. To facilitate the transition of fosfomycin to broader indications, including pneumonia, the National Institute of Allergy and Infectious Diseases (NIAID) initiated a Phase 1 clinical study on the safety and intrapulmonary pharmacokinetics (PK) of fosfomycin in healthy volunteers, in which samples were collected from not only plasma but also from two main sites of infection, namely pulmonary epithelial lining fluid (ELF) and alveolar macrophages (AMs); results from the original study have been recently published in *Antimicrobial Agents and Chemotherapy* (15)

The precious intrapulmonary PK data of fosfomycin provide an opportunity to quantitatively evaluate the relationship among different dosing regimens, drug exposure at the infection site, and the probability of target attainment (PTA) using a modeling approach. Antimicrobial resistance is associated with suboptimal dosing regimens that result in insufficient exposure at the infection sites. Since attaining drug concentrations above the MIC is crucial for bacterial eradication and preventing resistance, tailoring dosing regimens based on the concentrations at the infection site may lower the risk of developing antimicrobial resistance (16). In this study, we utilized fosfomycin PK data from plasma, ELF, and AMs to develop a minimal physiologically based pharmacokinetics (m-PBPK) model. This model enables us to evaluate the effects of pathophysiological changes that occur during pneumonia on drug concentrations at the infection site. Furthermore, we linked fosfomycin PK data to its antimicrobial efficacy (PD effect) by conducting a PTA analysis to evaluate the likelihood of achieving the desired PD effect across a wide range of MIC values following the administration of different dosing regimens (17). The PTA analysis helped us propose a dosing recommendation for pneumonia populations that can be utilized in subsequent clinical studies and future clinical practice.

## MATERIALS AND METHODS

### Data source

**Fig 1:**
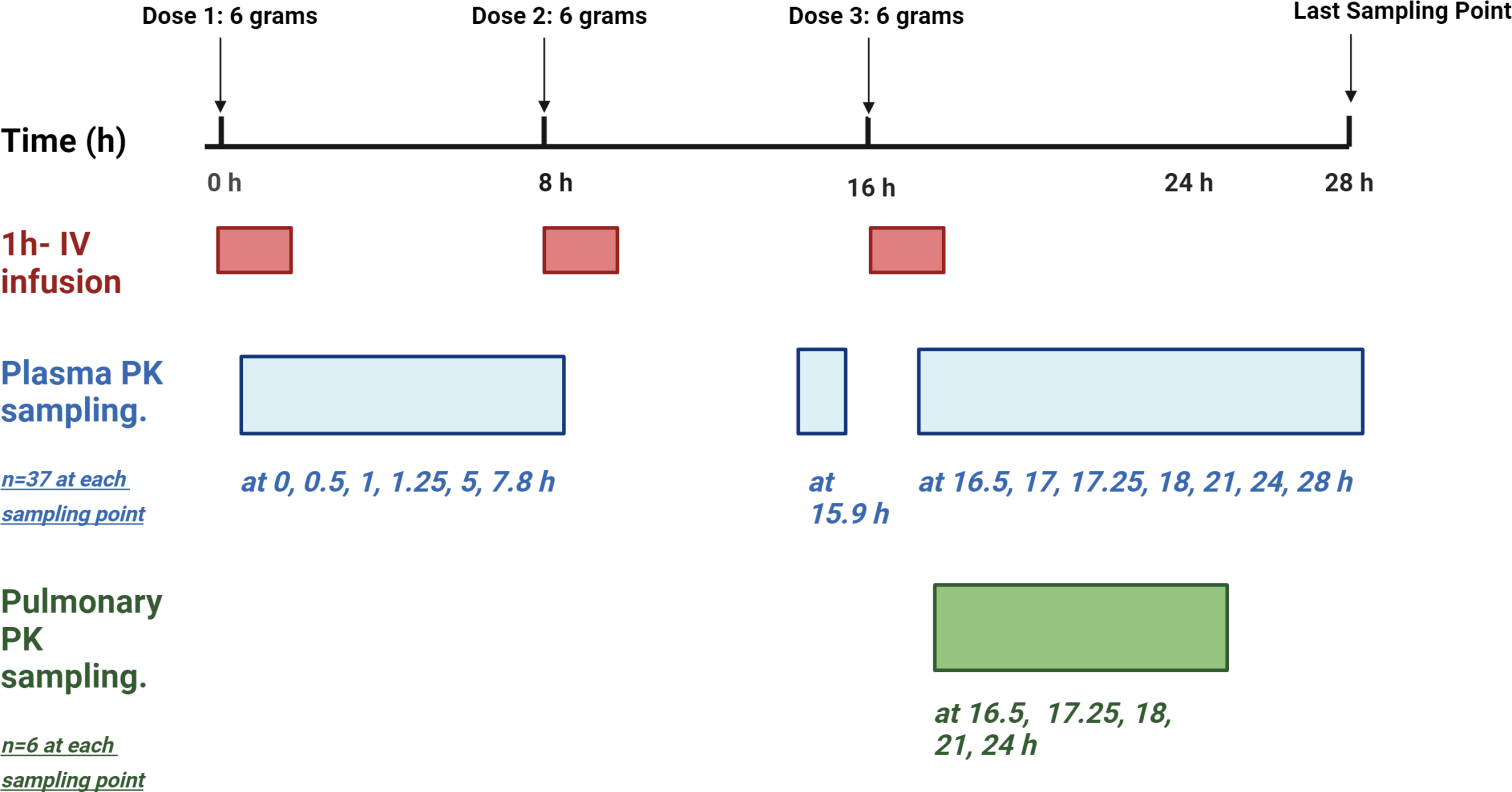
Schematic diagram of the study design. The figure was created using BioRender.

The data were obtained from the NIAID Phase 1 clinical study (NCT03910673) conducted at the Duke University Early Phase Clinical Research Unit (DEPRU) after being approved by the university’s institutional review board. The study investigated the safety and intrapulmonary PK of fosfomycin administered by intravenous injection (ZTI-01) in healthy participants. After obtaining written informed consent, 37 participants aged 18–45 years with normal renal function were enrolled to receive three doses of 6 g intravenous fosfomycin infused over 1 h every 8 h (q8h). Blood samples were collected before the administration of all doses and at 0.5, 1, 1.25, 2, and 5 h after the administration of the first and third doses, with two more sampling points collected at 8 and 12 h after the third dose. Blood samples were centrifuged at 2,000 *g* for 15 min, and plasma samples were then collected and stored at −70°C or below. Bronchoscopy with bronchoalveolar lavage (BAL) was performed at randomly assigned time points (0.5, 1.25, 2, 5, and 8 h) after the third dose from 30 participants. Each participant contributed to one BAL sampling time point, with six participants per time point. A detailed schematic diagram of the study is presented in Fig. 1. Concentrations of fosfomycin in ELF and AM cells from BAL samples, as well as in plasma samples, were analyzed using validated assay of liquid chromatography with tandem mass spectrometry (LC-MS/MS).

### Model development

Using data from these participants, population PK model (compartmental and m-PBPK) for plasma, ELF, and AMs were developed for fosfomycin using the nonlinear mixed effect model program NONMEM (Version 7.4.3; Icon Development Solutions, Ellicott City, Maryland) interfaced with Pirana (Version 2.9.9). The first-order conditional estimation method with interaction (FOCEI) and a user-defined subroutine (ADVAN6 or ADVAN8) were used to estimate the PK parameters, interindividual variability (IIV), and residual variability (RV). All the data, including plasma, ELF, and AMs were analyzed simultaneously, and the IIV was estimated using an exponential model, while the RV was modeled with a proportional error model. Data processing and graphical analysis were performed using RStudio 4.2.2 (RStudio4, PBC, Boston, Massachusetts) and GraphPad Prism (Version 10.0.2; La Jolla, California, United States).

#### Compartmental model

The compartmental model was initially developed to characterize fosfomycin intrapulmonary PK. The detailed development history of the compartmental model is presented in Table S1 in the supplementary material. The final compartmental model structure, shown in Fig. S1 in the supplementary material, consists of four compartments: central, peripheral, ELF, and AMs. The final estimated parameters are presented in Table S2 in the supplementary material. Moreover, the goodness-of-fit plots in Fig. S2 demonstrate that the model accurately captures drug concentrations in plasma, ELF, and AMs with minimal bias. Given the limitations of compartmental models in representing the complexities of physiological processes and anatomical structures, we transitioned to a m-PBPK model.

#### m-PBPK model

##### Base model

**Fig 2:**
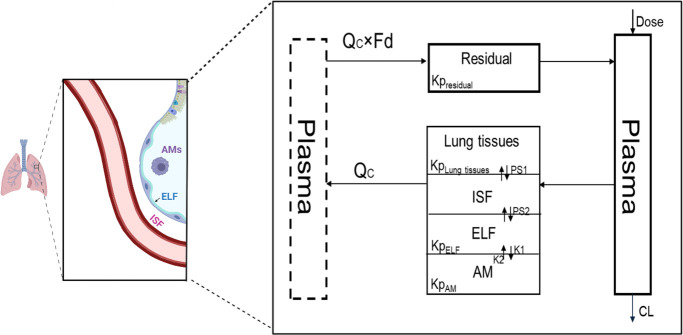
The final proposed m-PBPK model structure following the administration of IV-infused fosfomycin includes plasma, lung (comprising lung tissues, interstitial fluid [ISF], epithelial lining fluid [ELF], and alveolar macrophages [AMs]), and residual (includes all the other organs). The plasma compartment (dashed line) on the left is identical to the plasma compartment on the right, and the lung receives full cardiac output (Q_C_), while the tissues receive a fraction (*fd*). The figure was created using BioRender.

The proposed m-PBPK model consists of three main compartments: plasma, lung, and residual tissues, where all other organs are lumped into a single compartment. The lung compartment is further subdivided into four distinct compartments: lung tissues, interstitial fluid (ISF), ELF, and AMs. The ELF is a thin aqueous layer that covers the alveolar surfaces, while the AMs are the main immune cells found within the ELF space, accounting for nearly 80% of all immune cells in this fluid (18). This model, as shown in Fig. 2, is established based on the anatomical structure of the lung. When fosfomycin enters the lung, it first distributes into the ISF. From there, it is further distributed to the lung tissues and the ELF. Once in the ELF, fosfomycin can enter the AMs through an uptake mechanism. Like all the m-PBPK models, the proposed model has some physiological constraints: *fd* (fraction of the cardiac output) ≤1, and the total volume of all compartments equals the body weight. Additionally, the parameter *fd* × Q_C_ represents distribution clearance (19). Physiological parameters, such as cardiac output (Qc), lung volume (V_Lung_), plasma (V_p_), ISF (V_ISF_), ELF (V_ELF_), and AMs (V_AMs_) were fixed to their reported physiological values (18–23). The fixed parameters are listed in Table 1.

**TABLE 1:** Physiological parameters used in the development of m-PBPK (for a 70 kg human)

Parameter (unit)	Definition	Value	Reference
Q**_C_** (L/hr)	Cardiac output	336	(19)
Vp (L)	Plasma volume	3	(19, 20)
V**_residual_** (L)	Human tissue volume	66	(19)
V_ISF_ (L)	Interstitial fluid volume	0.3	(22)
V_ELF_ (L)	Epithelial lining fluid volume	0.026	(18, 21, 23)
V_AMs_(L)	Alveolar macrophages volume	0.0026	(18)
V_Lung_ (L)	Volume of the lung tissue	0.7	(20)

##### Base model limitation and further modifications performed

Initially, we did not include a partition coefficient for the ELF (Kp_ELF_) in our model because fosfomycin has negligible protein binding (13), and the protein level in the ELF space is much lower compared with the plasma (18). Therefore, we assumed that drug distribution in the ELF occurred without significant binding. While the base model accurately captured fosfomycin PK in plasma, it did not adequately capture drug concentration in the ELF and AMs. To address this, we conducted further literature research and revised our model based on our improved understanding of the ELF composition. We learned that surfactants are the primary components of the ELF, with phosphatidylcholine accounting for approximately 80% of the total surfactant content (its structure is shown in Fig. S3 in the supplementary material) (24). The polar head of this surfactant contains a negatively charged phosphate group and a positively charged quaternary amine. Since fosfomycin will be negatively charged in the pulmonary region, where the pH is approximately 7.4 in the ISF and 6.6 in the ELF (23), and considering its pKa value of 1.25 (25), we added the partition coefficient in the ELF (kp_ELF_) to account for the binding interaction between fosfomycin and surfactant.

The addition of the Kp_ELF_ parameter significantly enhanced the model’s ability to predict drug concentration in the ELF. Accordingly, Kp_ELF_ was incorporated in the final model.

##### Final model

The equations that describe the rate of fosfomycin transfer between the compartments in our final m-PBPK model are listed below:


(1)dA plasmadt=Qc×(AISFVISF−APlasmaVp)+Fd×Qc×(AresidualVresidual×Kpresidual−AplasmaVp)−CL×AplasmaVpA plasma(0)=0(2)dAISFdt=Qc×(AplasmaVp−AISFVISF)+PS1×(Alung, tissueVLung×Kplung tissues−AISFVISF)−PS2×(AISFVISF−AELF VELF×KpELF)AISF(0)=0(3)dAlung,tissuesdt=PS1×(AISFVISF−Alung tissuesVLung×Kplung tissues)Alung tissues(0)=0(4)dAELFdt=PS2×(AISFVISF−AELFVELF×KpELF)−K1×AELFKpELF+K2×AAMKpAMAELF(0)=0(5)dAAMdt=K1×AELFKpELF−K2×AAMKpAMAAM(0)=0(6)dAresidualdt=Fd×Qc×(APlasmaVp−Aresidual Vresidual×Kpresidual)Aresidual(0)=0.

In the equation above, the variables are presented in the general format of A_compartment_, where A represents the amount, and Kp_compartment_, where Kp represents the partition coefficient. The Kp is defined as the ratio of drug concentrations between a tissue (or compartment) and plasma. PS refers to the permeability-surface product, either between the ISF and lung tissue (PS_1_) or between the ISF and ELF (PS_2_). Cardiac output is represented by (Qc), while K_1_ and K_2_ are the uptake and efflux rate constants, respectively. The names and definitions for all PK parameters are provided in Table 2.

**TABLE 2:** Estimated population parameters from the final m-PBPK model

Parameter (unit)	Definition	Estimate (RSE%)	Shrinkage
Fd (unitless)	Fraction of the cardiac output with Fick’s law of perfusion	0.396 (2.8%)	
Kp_Lung tissues_ (unitless)	Partition coefficient in the lung tissue	11.6 (6.6%)	
Kp_residual_ (unitless)	Partition coefficient in the tissues	0.142 (7.8%)	
Kp_AM_ (unitless)	Partition coefficient in the AM	0.000023 (9.1%)	
Kp_ELF_ (unitless)	Partition coefficient in the ELF	0.393 (5.1%)	
PS_1_ (L/h)	Permeability surface product (between ISF and lung tissue compartment)	4.55 (4.3%)	
PS_2_ (L/h)	Permeability surface product (between ISF and ELF)	0.0316 (52.5%)	
K_1_ ($h{}^{-1}$)	Uptake rate constant	0.00515 (11.9%)	
K_2_($h{}^{-1}$)	Efflux rate constant	0.000006 (32.0%)	
CL (L/h)	Systematic clearance	7.0 (1.9%)	
IIV- CL (%)	Interindividual variability on CL	12.1% (4.0%)	3.0%
IIV- K_2_ (%)	Interindividual variability on K_2_	40.0% (9.0%)	20.0%
σ^2^ (Proportional)	Residual variability	0.0303 (3.0%)	5.0%

### Model evaluation

The final selection of the model was determined based on its biological and physiological relevance, quality of goodness-of-fit plots, stability of parameter estimates, and objective function value (OFV). For comparing the models, the likelihood ratio test was employed, requiring a decrease of 3.84 points in the NONMEM objective function (OFV= −2 log likelihood) to demonstrate a statistically significant improvement in model performance (statistically significant at α = 0.05).

### Sensitivity analysis

The model was initially developed based on healthy participants. However, during the infection, various pathophysiological changes may occur which could potentially affect the model parameters. These pathophysiological changes include (i) the permeability of the lung membrane is often increased (26), which can result in an increase in PS_1_ and PS_2_, (ii) the number of alveolar macrophages will decrease, as the neutrophil will be the predominant innate immune cell leading to a reduction in V_Ams_ (27), (iii) alveolar edema causes an increase in fluid in the alveolar space, resulting in an increase in V_ELF_ (3), and (iv) surfactant inactivation may also reduce the Kp_ELF_ value (28). Collectively, these changes may lead to significant alterations in drug concentrations at the infection site. To investigate how variations in these parameters affect drug concentrations in the ELF, we conducted a sensitivity analysis.

In the sensitivity analysis, we fixed all final PK parameters from the final model and physiological values obtained from healthy participants, then we modified one parameter at a time—PS_1_, PS_2_, V_AMs_, V_ELF_, and Kp_ELF_—to evaluate the effect of each individual change on the intrapulmonary PK of fosfomycin. The adjusted values for each parameter are provided in Table 3. We conducted the sensitivity analysis to predict the PK of fosfomycin at the infection site in pneumonia patients. This step is essential for our next PTA analysis as it enabled us to propose a dosing regimen for the patient population, considering various pathophysiological conditions and their impact on drug concentration at the infection site, specifically in the ELF. We investigated the effect of changing the following parameters on the drug concentration in the ELF space:

**TABLE 3:** Comparison of the values of some PK parameters estimated from the final model and modified model parameters to account for pathophysiological changes during infection and their impact on fosfomycin ELF PK profile

Parameter (unit)	Value in the final model in the healthy population	Value in the modified model in pneumonia patient population	Impact on the drug concentration in the ELF
PS_1_ (L/h)	4.55	11.2	Minor decrease in the drug concentration in the ELF
PS_2_ (L/h)	0.0316	0.079	Minor increase in the drug concentration in the ELF
PS_1_, PS_2_ (L/h)	4.55, 0.0316	11.2, 0.079	Negligible effect
V_AMs_ (L)	0.0026	0.00065	No effect
V_ELF_ (L)	0.026	0.039	The drug concentration in the ELF decreases
Kp_ELF_ (unitless)	0.393	0.29	The drug concentration in the ELF decreases

#### Increasing membrane permeability (increasing the PS_1_ and PS_2_)

Increasing alveolar membrane permeability is due to the elastase that is produced by neutrophils, which causes proteolysis of pulmonary tissue. Plasma elastase levels correlate significantly with the pulmonary vascular permeability index, which increases 2.5-fold in pneumonia patients (26). Consequently, we assumed a 150% increase in the permeability surface area product (PS) parameter.

#### Increasing the ELF volume (increasing V_ELF_)

No specific data are available on the extent of ELF volume increase in bacterial pneumonia. However, mild intra-alveolar edema is known to develop in the early stages of infection (3). In the absence of quantitative data, we assumed a 50% increase in ELF during infection. This assumption has been applied in previous studies to quantify pathophysiological changes and their impact on the PK profile of antibiotics at the infection site (23).

#### Changing the cell population (decreasing V_AMs_)

In a healthy population, the percentage of AMs in the BAL samples is over 80% (18, 29). However, this percentage decreases to approximately 20% in patients with pneumonia (27). Therefore, we assumed a 75% reduction in the V_AMs_ value during infection. The adjusted value was set to 0.00065 L compared with the initial value of 0.0026 L.

#### Inactivation of the surfactant (decreasing Kp_ELF_)

During infection, surfactant homeostasis is disrupted (2). Increased membrane permeability leads to higher albumin concentrations in the pulmonary region, which can deactivate the available surfactant (28). Specific quantitative data on surfactant levels in adult pneumonia patients are lacking. Therefore, we used plasma albumin levels as a surrogate marker, as many studies have reported that hypoalbuminemia is highly associated with pneumonia severity (30). Albumin levels can drop rapidly during acute infections due to increased membrane permeability, which results in the leakage of albumin from the bloodstream into the surrounding tissues (31). In a healthy population, normal albumin levels range from 3.5 to 4.5 g/dL (32), and the Kp_ELF_ was estimated to be 0.393. In pneumonia patients, mild hypoalbuminemia can occur, leading to a 26% decrease in albumin levels, which lowers them to a range of 2.5 to 3.5 g/dL (32). We applied this same percentage decline to Kp_ELF_, resulting in a new value of 0.29.

### Probability of target attainment (PTA) analysis

Based on the final PK parameters presented in Table 2, we conducted a Monte Carlo simulation in NONMEM (Version 7.4.3) to evaluate the PTA of fosfomycin in plasma and ELF following the administration of several dosing regimens, including 4, 5, 6, or 8g q8h, and 8 or 10 g q12h, each infused over 1 h. All the evaluated dosing regimens are within the approved daily doses (33, 34). For the pneumonia population, we adjusted the PK parameters from the final model to account for the impact of pathophysiological changes that occur during pneumonia based on the findings from our sensitivity analysis. Specifically, we set Kp_ELF_ to 0.29 and V_ELF_ to 0.039 L, as the analysis indicated that these adjustments would lower drug concentrations in the ELF, while plasma concentrations would remain unchanged. Subsequently, we evaluated the PTA% of fosfomycin in this population after the administration of the same dosing regimens.

For each scenario (healthy and pneumonia populations), we simulated 1,000 virtual subjects and generated concentration–time profiles in both plasma and ELF at steady state. AUC_24_ was calculated to predict the PTA% using PK/PD targets of AUC_24_/MIC 28.2 for *P. aeruginosa* and ≥42.8 for *S. aureus* (35, 36). The MIC values covered in our analysis ranged from 0.5 to 512 mg/L, consistent with previously reported values for fosfomycin against these two pathogens (37). Although *A. baumannii* can also cause HAP, no PK/PD target has been established for this organism; therefore, it was not included in our PTA analysis. Target attainment was considered successful if the PTA% was ≥90%. Additionally, for each pathogen and dosing regimen, we calculated the breakpoint, which is the highest MIC for which the PTA% reached at least 90%.

## RESULTS

### Estimated parameters

The final estimated parameters are presented in Table 2. The systematic clearance was estimated to be 6.59 L/h, consistent with the reported values (13). Furthermore, the permeability surface area product between the ISF and ELF (PS_2_) was estimated at 0.0316 L/h, which aligns with our expectations, as the alveolar membrane composed of tight junctions requires the drug in the ISF to penetrate the membrane to reach the ELF (18). The estimated partition coefficient in the lung tissues (Kp_Lung tissue_) is high, suggesting that the concentration of fosfomycin in homogenized lung tissue would be significantly higher than that in plasma. All estimated values show low relative standard error (RSE) values (less than 55%), and there is a small degree of shrinkage in the inter-variability (IVV), indicating the model’s reliability.

### Goodness of model fitting

**Fig 3:**
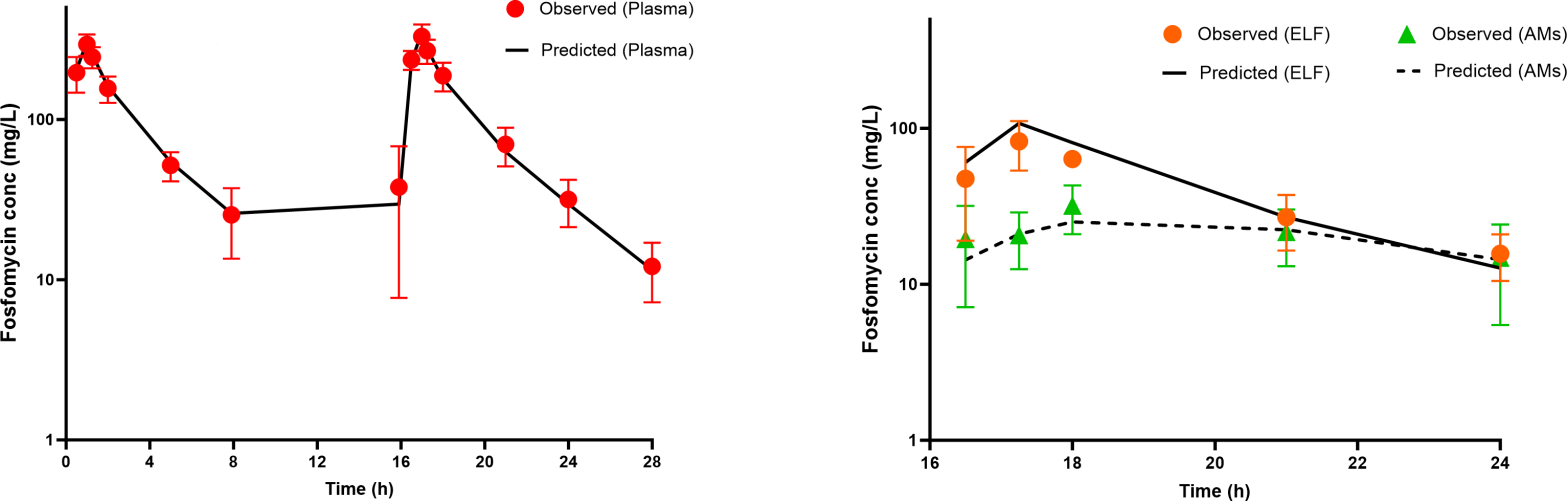
Observed (symbol) and model-predicted (line) concentration–time profiles for fosfomycin in (a) plasma (*n* = 37), (b) epithelial lining fluid (ELF), and (c) amacrophages (AMs). *n* = 6 at each sampling point in the ELF and AMs.

**Fig 4:**
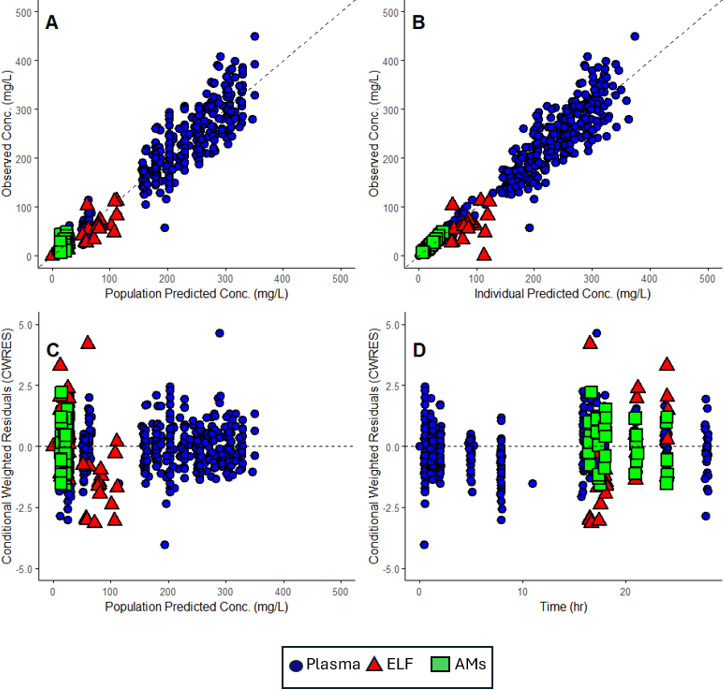
Goodness-of-fit plots for the population PK model of fosfomycin. (**A**) Observed versus population-predicted fosfomycin concentrations, (**B**) observed versus individual-predicted fosfomycin concentrations, (**C**) conditional weighted residuals versus population-predicted fosfomycin concentrations, (**D**) conditional weighted residuals versus time. Dotted black lines represent lines of identity in (**A**) and (**B**) and zero line in (**C**) and (**D**).

Figure 3 represents the time course of observed versus predicted concentrations in the plasma, ELF, and AMs. As shown in the figure, almost all the predicted concentrations fell within the standard deviation of the observed values. Additional diagnostic goodness-of-fit plots are shown in Fig. 4. As illustrated, the data are distributed around the line of identity or the zero-line, indicating that the model accurately captures the fosfomycin PK profile with minimal bias.

### Sensitivity analysis

**Fig 5:**
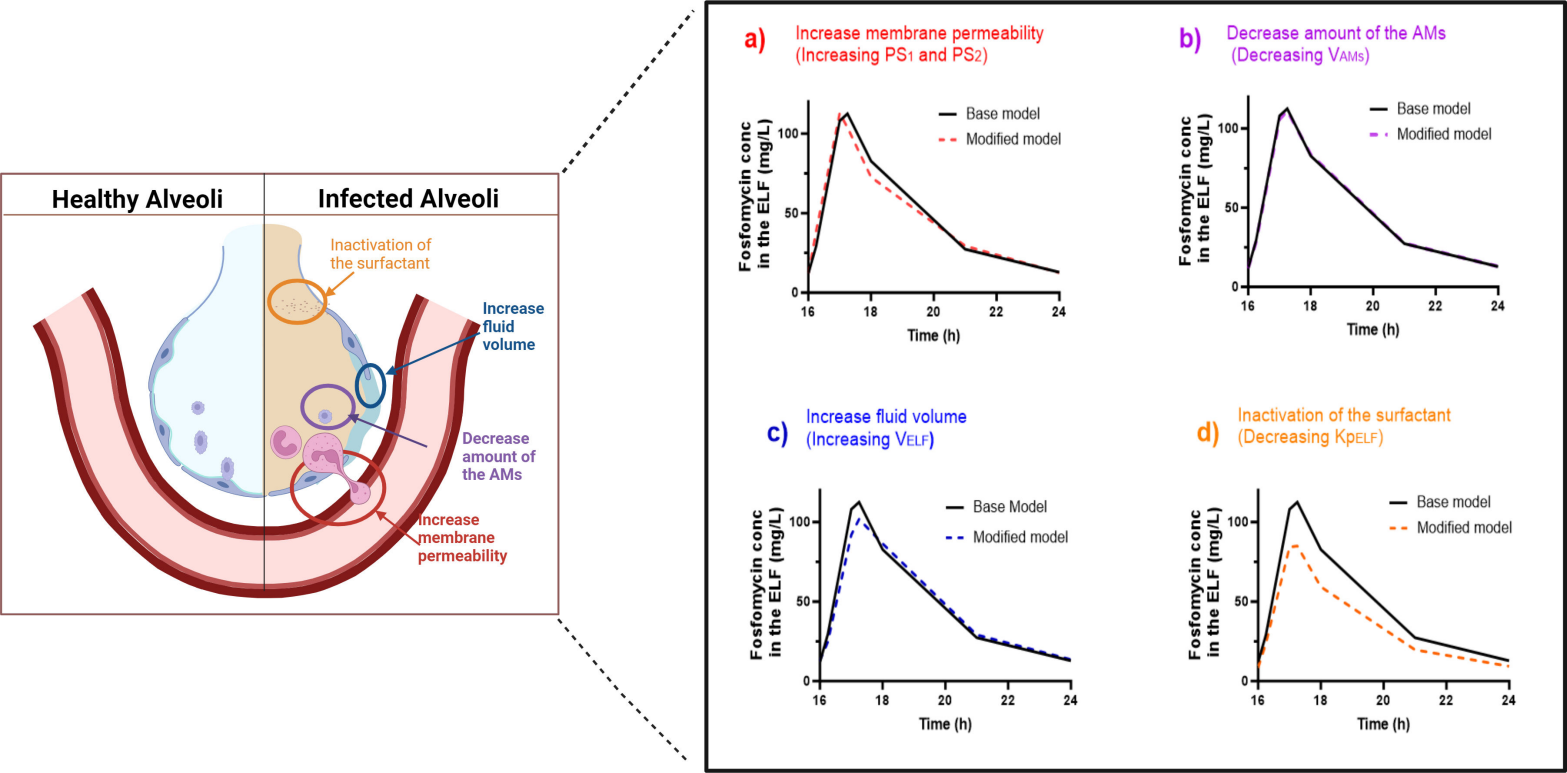
Pathophysiological changes during pneumonia and their impact on the PK profile in the ELF. (**a**) Increasing PS_1_ and PS_2_ due to enhanced membrane permeability lead to minor changes in the PK profile. (**b**) A decrease in the number of AMs, which reduces V_AMs_, does not alter the PK profile. (**c**) Increasing V_ELF_ due to alveolar edema leads to a minor reduction in drug concentration in the ELF. (**d**) Decreasing Kp_ELF_ due to surfactant deactivation results in a reduction in ELF concentration. The solid line represents the base model, while the dashed line represents the modified model due to the pathophysiological conditions. The figure was created using BioRender.

ELF and AMs are the main sites of infection for extracellular and intracellular pathogens, respectively (18). Intracellular pathogens can survive within host cells, including the AMs, requiring antibiotics to penetrate these cells for effective bacterial eradication. In contrast, extracellular pathogens cannot survive inside cells and are eliminated after being engulfed by the immune cells. Since both *P. aeruginosa* and *S. aureus* are classified as extracellular pathogens (38), we evaluated the effect of pathophysiological changes on drug concentration in the ELF, the main site for extracellular infection. The results showed that not all changes influence drug concentration in the ELF. Table 3 summarizes the modified model parameter values and their effects on the overall ELF PK profile. Moreover, Fig. 5 illustrates the pathophysiological changes associated with each parameter and their effect on the overall ELF PK profile. Increasing membrane permeability, which increases PS_1_ and PS_2_, and a decrease in the amount of AM in the alveolar space, which leads to a reduction in V_AM_, have no significant impact on the overall PK profile in the ELF, as shown in Fig. 5a and b, respectively. On the other hand, alveolar edema, which increases alveolar fluid and may raise V_ELF_ by 50%, was predicted to affect fosfomycin PK in ELF, albeit in a minor degree (Fig. 5c). In contrast, inactivating the surfactant, which results in a decrease in Kp_ELF_ by 26%, has the greatest impact on lowering drug concentrations in the ELF space, as illustrated in Fig. 5d. Consequently, we adjusted the values of V_ELF_ and Kp_ELF_ value in our PTA analysis to account for these two significant pathophysiological changes in pneumonia patients. None of these changes impact the drug concentration in plasma, as plasma concentration is primarily determined by other PK parameters, as previously described in Equation 1.

### Probability of target attainment (PTA) analysis

Based on the PK parameters estimated from the final model, a comprehensive Monte Carlo simulation was conducted to evaluate the PTA of fosfomycin in plasma and ELF for healthy participants. For the pneumonia population, Kp_ELF_ and V_ELF_ values were adjusted to 0.29 and 0.039 L, respectively, to reflect the pathophysiological changes that happened during the infection, and another Monte Carlo simulation was performed to evaluate the PTA of fosfomycin in plasma and ELF for this population. We evaluated the PTA following the administration of several dosing regimens using two specific PK/PD targets of AUC_24_/MIC > 28.2 for *P. aeruginosa* and 42.8 for *S. aureus*. As mentioned earlier, *A. baumannii* was not included in our PTA analysis due to the lack of a PK/PD index for this pathogen. Since our analysis relies on pathogen-specific PK/PD indices, extrapolating our findings to *A. baumannii* is not currently feasible. However, if a PK/PD index is established in the future, our model can be used to determine the appropriate dose for treating HAP caused by *A. baumannii*.

#### PTA evaluation against *S. aureus* in healthy and pneumonia populations

**Fig 6:**
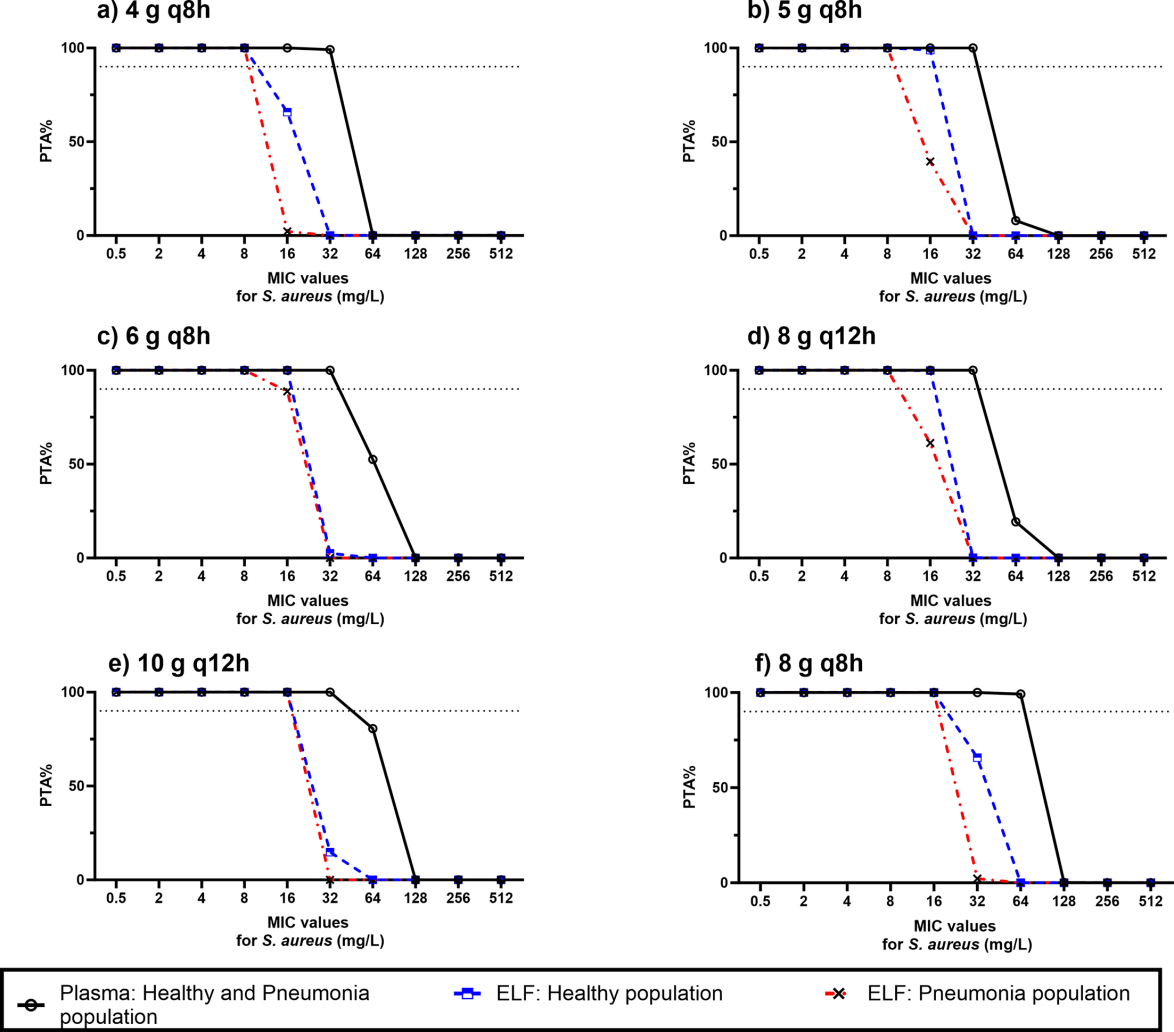
Comparison of PTA% of fosfomycin in plasma and ELF for healthy and pneumonia for fosfomycin versus MIC for *S. aureus* following the administration of (**a**) 4 g q8h, (**b**) 5 g q8h, (**c**) 6 g q8h, (**d**) 8 g q12h, (**e**) 10 g q12h, and (**f**) 8 g q8h. The dashed line represents ≥90% of the population who achieved the PK/PD target.

Figure 6 shows the PTA versus MIC profiles for various dosing regimens. PTA percentages in plasma for both healthy and pneumonia populations are higher than in ELF for healthy subjects and even more so than the ELF of pneumonia patients, especially at higher MIC values. For example, at MIC_90_ (8–64 mg/L), the concentration that inhibits 90% of the tested strains, the PTA in plasma after the administration of 8 g q8h is 100% compared with 0% in ELF in healthy and pneumonia populations. Despite this variation, all dosing regimens achieved 100% PTA in both plasma and ELF for both healthy and pneumonia populations at MIC_50_ (4 mg/L), the level that inhibits 50% of the tested strains. A heatmap of the PTA of fosfomycin over a wide MIC range, with six different dosing regimens in plasma and ELF for healthy and pneumonia populations, is shown in Table S3 in the supplementary material. Furthermore, Table 4 lists the breakpoint values that could be achieved following the evaluated dosing regimens. The breakpoints in plasma are consistently higher than those in the ELF for both healthy and pneumonia populations.

**TABLE 4:** Predicted fosfomycin breakpoints against several pathogens that caused pneumonia after the administration of different dosing regimens in healthy and pneumonia populations

Dosing regimen (infused over 1 h)	Pathogen	Predicted breakpoints (mg/L) in plasma or ELF		
		Plasma (healthy and pneumonia subjects)	ELF (healthy patients)	ELF (pneumonia patients)
4 g q8h	*P. aeruginosa*	32	16	16
	*S. aureus*	32	8	8
5 g q8h	*P. aeruginosa*	64	16	16
	*S. aureus*	32	16	8
8 g q12h	*P. aeruginosa*	64	16	16
	*S. aureus*	32	16	8
6 g q8h	*P. aeruginosa*	64	32	16
	*S. aureus*	32	16	8
10 g q12h	*P. aeruginosa*	64	32	16
	*S. aureus*	32	16	16
8 g q8h	*P. aeruginosa*	64	32	32
	*S. aureus*	64	16	16

#### PTA evaluation against *P. aeruginosa* in healthy and pneumonia populations

**Fig 7:**
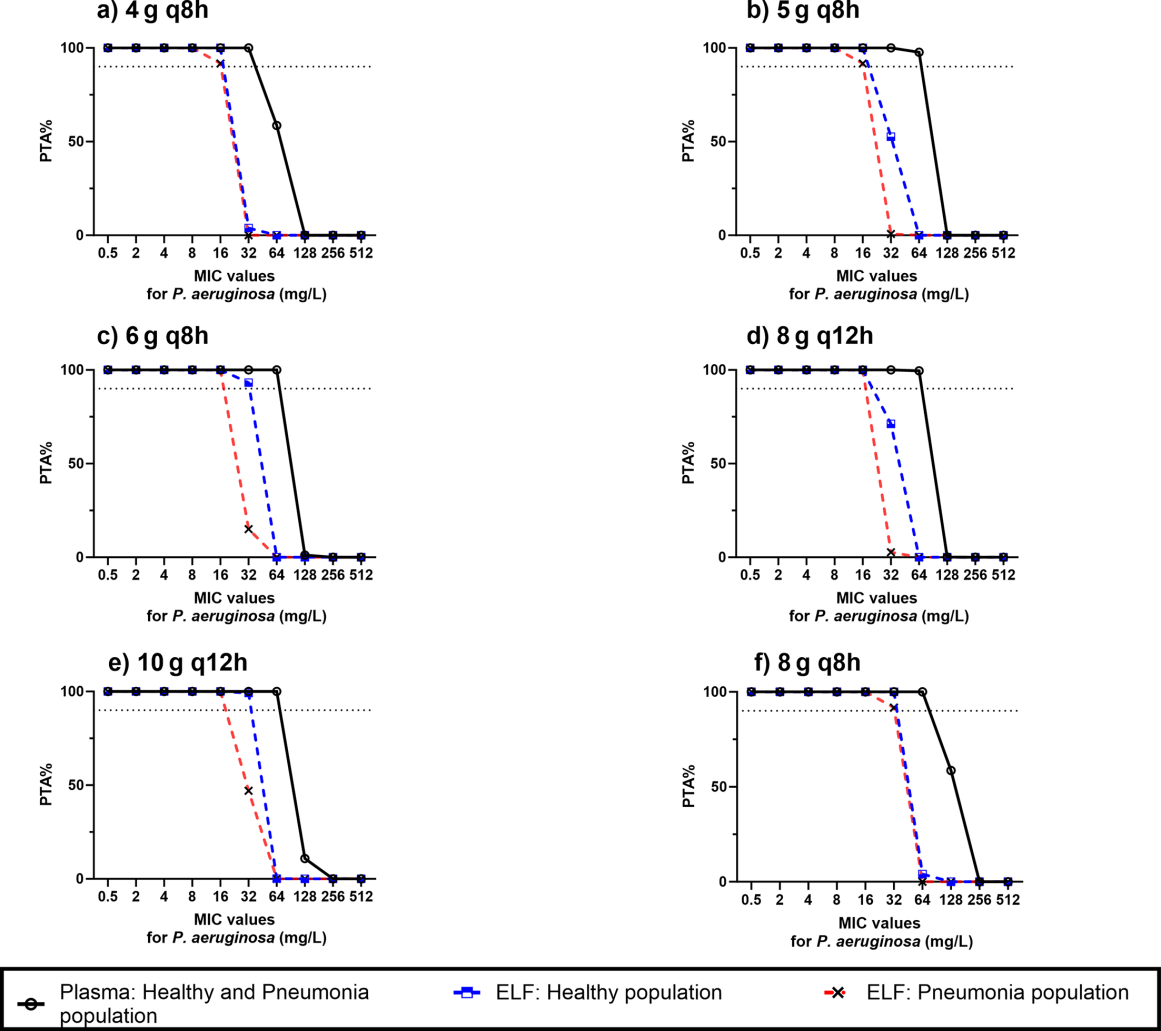
Comparison of PTA% of fosfomycin in plasma and ELF for healthy and pneumonia for fosfomycin versus MIC for *P. aeruginosa* following the administration of (**a**) 4 g q8h, (**b**) 5 g q8h, (**c**) 6 g q8h, (**d**) 8 g q12h, (**e**) 10 g q12h, and (**f**) 8 g q8h. The dashed line represents ≥90% of the population who achieved the PK/PD target.

Figure 7 shows the PTA versus MIC profiles for various dosing regimens. PTA percentages in plasma for both healthy and pneumonia populations are higher than in the ELF for healthy and pneumonia patients, even at MIC values below MIC_50_ (32–64 mg/L). For example, at an MIC_50_ of 32 mg/L, the PTA in plasma after administering 4 g q8h is 100%, while the PTA in ELF is approximately 0% for both healthy subjects and pneumonia patients. A heatmap of the PTA of fosfomycin over a wide range of the MIC values following the administration of six different dosing regimens in plasma and ELF for both healthy and pneumonia populations is presented in Table S3 in the supplementary material. Table 4 provides the breakpoint values that could be reached following the administration of the evaluated dosing regimens. As expected, the breakpoints in plasma are consistently higher than those in the ELF for both healthy and pneumonia populations. Additionally, none of the evaluated dosing regimens achieved the breakpoints equal to MIC_90_ (64–128 mg/L) in the ELF, while a dose of 5, 6, or 8 g q8h, and 8 or 10 g q12h—each infused over 1 h resulted in a breakpoint of 64 mg/L in plasma for both healthy and pneumonia populations.

## DISCUSSION

In this study, we successfully developed a m-PBPK model that adequately characterized the intrapulmonary PK of fosfomycin in humans. Although there are several modeling reports on the intrapulmonary PK of multiple antibiotics available in the literature (39–42), most of them focus on conventional compartment models that lack mechanistic insight, adaptability, and extrapolability. In contrast, our m-PBPK model was based on the anatomical structure of the lung, enhancing its physiological relevance. Due to its mechanistic and physiological nature, the PBPK model, including the m-PBPK model, can be extrapolated from one species to another species (e.g., interspecies scaling from animal to human), or different age groups within the same species (e.g., dose extrapolation from adult to infant), or from one population to another population, such as from healthy adults to pneumonia patients as we presented in the current study. Leveraging the advantage of an m-PBPK model, we were able to perform a sensitivity analysis to evaluate the impact of several pathophysiological conditions on the intrapulmonary PK of fosfomycin in the target population (i.e., patients with pneumonia), through which we gained a deeper understanding of fosfomycin exposure at the site of infection.

Our sensitivity analysis revealed that during infection, drug concentrations at the infection site decrease, whereas drug concentrations in plasma remain unchanged. This finding underscores the importance of assessing drug efficacy at the infection site rather than relying on plasma data alone, as plasma concentration may overestimate the drug’s effectiveness. For instance, in healthy participants, fosfomycin levels in the ELF are lower than in plasma due to restricted penetration across the alveolar membrane, primarily caused by tight junctions. Additionally, the hydrophilic nature of fosfomycin further limits its distribution into the ELF. During infection, ELF concentrations are expected to decline further due to pathophysiological changes, such as increased alveolar fluid volume, leading to drug dilution and surfactant deactivation, reducing drug partitioning into the ELF. The sensitivity analysis strengthened our understanding of fosfomycin’s intrapulmonary PK during infection and enabled us to propose a dosing regimen tailored for pneumonia patients by incorporating the pathophysiological changes that occur during infection. As such, it was instrumental in evaluating the PTA% for fosfomycin in both plasma and ELF for healthy individuals and pneumonia patients.

While we recognize the significance of drug concentration at the infection site, it’s still unclear if they truly reflect fosfomycin effectiveness against the main HAP pathogens. Currently, no PK/PD indices for fosfomycin derived from ELF concentrations have been established using an *in vivo* pneumonia infection model. Fosfomycin’s antimicrobial efficacy against HAP pathogens has primarily been assessed through PK/PD indices derived from *in vitro* models or plasma measurements in *in vivo* thigh infection models (35, 36). Although these models provide valuable insights, they may not fully capture the unique anatomy and physiology of the lungs. Ideally, PK/PD relationships for fosfomycin should be investigated using an *in vivo* pulmonary infection model to derive a PK/PD index directly from the infection site. Such data would be crucial for accurately establishing dosing recommendations for clinical studies.

Our PTA results indicate that for treating HAP caused by *P. aeruginosa*, if plasma concentrations are the most reliable indicator of fosfomycin’s antimicrobial efficacy, all evaluated dosing regimens successfully achieved target attainment at MIC values up to the MIC_50_ for *P. aeruginosa* (32–64 mg/L), particularly at 32 mg/L. This suggests that a dose of 4 g q8h administered as a 1 h infusion could be considered as an appropriate therapy for achieving effective plasma concentrations in both healthy and pneumonia populations. However, if drug concentrations in the ELF are the more accurate predictor of therapeutic efficacy, a higher dose, specifically, 6 g q8h administered over a 1 h infusion, would be required to achieve comparable target attainment. Due to the expected reduction in ELF concentrations in pneumonia patients, even higher doses might be needed in this population. Specifically, we recommend a dose of 8 g q8h administered over a 1 h infusion to achieve sufficient target attainment at MIC values up to 32 mg/L.

Determining an appropriate dose regimen for HAP caused by *S. aureus* was more straightforward, as there was consistency between the PTA percentages in both plasma and ELF for healthy individuals and pneumonia patients at MIC values up to the MIC_50_ (4 mg/L). As a result, a dose of 4 g q8h, infused over 1 h could be administered to achieve adequate concentrations in both plasma and ELF in these populations.

Although we evaluated a wide range of MIC values, we primarily focused on MIC_50_ for dosing recommendations. For institutions with their own fosfomycin MIC distribution data, our PTA results can serve as a useful reference to guide physicians in selecting appropriate dosing regimens for different stages of pneumonia. To facilitate these dosing decisions, we provided a heatmap (Table S3) illustrating the PTA of fosfomycin in plasma and ELF for healthy and pneumonia populations at different MIC values after the administration of various dosing regimens. Moreover, we calculated the predicted breakpoints (summarized in Table 4) for each pathogen after the administration of several dosing regimens. Together, these resources aim to enhance the effectiveness of fosfomycin therapy in clinical practice.

Our work has several limitations, primarily related to assumptions made in the sensitivity analysis, which require further validation. First, we assumed a 50% increase in ELF volume (V_ELF_) in pneumonia patients, although the actual extent of this increase is currently uncertain. Second, we used plasma albumin concentration, instead of surfactant level, to estimate changes in Kp_ELF_; while this may not be ideal, we consider it a reasonable surrogate marker since hypoalbuminemia has been found to be linked to infection severity (30). It’s important to note, however, that hypoalbuminemia can also result from physiological changes, such as those occurring during pregnancy (43), and other pathophysiological conditions like liver cirrhosis (44). Nevertheless, our m-PBPK model is adaptable. For institutions that have their in-house ELF volume and surfactant concentration data for pneumonia patients, they can easily substitute their values to evaluate the impact of these pathophysiological conditions on the ELF PK profile. Additionally, due to data limitations, we did not evaluate the impact of renal impairment on fosfomycin PK. Considering that fosfomycin is primarily eliminated through the kidneys, our current dosing recommendations should be adapted with caution for pneumonia patients with severe renal impairment.

While this work proposes a dosing regimen for the subsequent clinical study, future work will include validating the model with patient data to confirm its predictive performance and refine dose recommendations if needed. Furthermore, the proposed model could be extended to optimize dosing in patients with community-acquired pneumonia (CAP), as fosfomycin is effective against the primary pathogens causing CAP, thus broadening its applicability beyond HAP. An important next step will also involve quantifying additional pathophysiological changes in critically ill patients, as these alterations can significantly impact fosfomycin PK. Fluid resuscitation and capillary leak may increase the volume of distribution, leading to lower plasma drug concentrations. Renal impairment or augmented renal clearance (ARC) can reduce or enhance fosfomycin elimination, respectively. Septic shock can further complicate drug distribution by initially increasing cardiac output, followed by a decline (45). Although these factors were not evaluated in the current study, they may substantially affect fosfomycin PK and safety in this vulnerable population. Future work will aim to incorporate these clinical complexities to better inform dosing strategies for this special population.

In conclusion, we successfully developed the m-PBPK model to predict fosfomycin concentrations in plasma, ELF, and AMs, allowing us to assess the impact of pneumonia on drug concentrations at the infection site and propose dosing regimens. While it remains uncertain regarding whether plasma or ELF concentrations are more predictive of efficacy or if our assumptions regarding pathophysiological changes are entirely accurate, we proposed different dosing regimens after evaluating the PTA% in plasma and ELF in healthy and pneumonia populations. If plasma concentration is the appropriate efficacy indicator, then a dose of 4 g q8h should be used for *S. aureus* and *P. aeruginosa* infections. However, if ELF concentration is the more accurate indicator, the same dose could be applied for treating *S. aureus* infections in both healthy individuals and those with pneumonia. In the case of *P. aeruginosa* infections, a dose of 6 g q8h infused over 1 h is recommended. Moreover, considering the potential for reduced drug penetration in pneumonia patients, higher doses—specifically, 8 g q8h infused over 1 h—could be considered for this population.

## SUPPLEMENTAL MATERIAL

Supplemental material aac.01869-24-s0001.docx Figures S1 to S3; Tables S1 to S3.

ASM does not own the copyrights to Supplemental Material that may be linked to, or accessed through, an article. The authors have granted ASM a non-exclusive, world-wide license to publish the Supplemental Material files. Please contact the corresponding author directly for reuse.
