# Supplementary material for: Determining an appropriate fosfomycin (ZTI-01) dosing regimen in pneumonia patients by utilizing minimal PBPK modeling and target attainment analysis
Source: Antimicrob Agents Chemother. 2025 May 5;69(6):e01869-24. doi: 10.1128/aac.01869-24 (PMC12135528; doi:10.1128/aac.01869-24)
Supplement: Supplemental material — Figures S1 to S3; Tables S1 to S3. [file aac.01869-24-s0001.docx]

**Supplementary File**

**Determining an Appropriate Fosfomycin (ZTI-01) Dosing Regimen in Pneumonia Patients by Utilizing minimal PBPK Modeling and Target Attainment Analysis**

**Authors:** Jomana Al Hroot^1^, Joshua Reeder^1^, Xuanzhen Yuan^1^, Kenan Gu^2^, Emmanuel Walter^3,4^, Lindsay Boole^5^, Loretta Que^5^, Guohua An^1^*.

^1^ Department of Pharmaceutical Sciences and Experimental Therapeutics, College of Pharmacy, University of Iowa, Iowa city, IA, USA

^2^ Division of Microbiology and Infectious Diseases, National Institute of Allergy and Infectious Diseases, Rockville, MD, USA.

^3^ Duke Human Vaccine Institute, Duke University School of Medicine, Durham, NC, USA

^4^ Department of Pediatrics, Duke University School of Medicine, Durham, NC, USA

^5^ Division of Pulmonary, Allergy, and Critical Care Medicine, Duke University of School of Medicine, Durham, NC, USA

_*_**Corresponding Author:** Guohua An, MD, PhD,

Department of Pharmaceutical Sciences and Experimental Therapeutics, College of Pharmacy, University of Iowa, 115S Grand Ave, Iowa City, IA 52242.

E-mail: [guohua-an@uiowa.edu](mailto:guohua-an@uiowa.edu)

**Final Compartmental model Structure**

**Table S1 The compartmental model development history**

| Model No | Used data | Model structure | Model Description | OFV |
| --- | --- | --- | --- | --- |
| 1 | Only Plasma | Two compartmental model | In the two- compartmental model, one compartment (Central), and another compartment (Peripheral). The elimination from the central compartment. The IVV was on the volume of the central and Peripheral compartment and CL. | 3369.42 |
| 2 | Plasma and Epithelial Lining Fluid (ELF) | Three compartment model | In the three-compartmental model, the volume of the ELF was estimated. The elimination from the central compartment. The drug first reaches the central compartment, then it distributes to the peripheral and the ELF compartment. The IVV was on the volume of the central compartment and CL. | 3602.841 |
| 3 | Plasma and ELF | Three compartment model | In the three compartmental model, the volume of the ELF was fixed to its physiological value (0.026 L). The elimination from the central compartment.  The drug first reaches the central compartment then it distributes to the peripheral or the ELF compartment. The IVV was on the volume of the central compartment and CL. | 3623.632 |
| 4 | Plasma, ELF, and Alveolar Macrophages (AMs). | Four compartmental model | In the four compartmental model, the volume of the ELF and AM were estimated values. The elimination from the central compartment.  The drug first reaches the central compartment then it distributes to the peripheral and the ELF compartment, and from the ELF it will go back to the central and distribute to the AMs. The IVV was on the volume of the central compartment and CL. | 3820.277 |
| 5 | Plasma, ELF, and AMs. | Four compartmental model | In the four compartmental model, the volume of the ELF and AM were fixed to their physiological value (0.026 L and 0.0026 L, respectively). The elimination from the central compartment. The drug first reaches the central compartment then it distributes to the peripheral and the ELF compartment, and from the ELF it will go back to the central and distribute to the AMs.  The IVV was on the volume of the central compartment and CL. | 3851.828 |

Figure 1S: Model structure of fosfomycin using plasma, ELF, and AM samples. Q represents the distribution flow between the compartments, k13 and K31 represents the uptake and efflux rate constant, K34 and K43 represent the AM uptake and efflux rate constant, respectively. The volume of the ELF and AM compartments were either estimated in Model 4 or fixed in Model 5.

**Table S2: Final population PK model parameter estimates for fosfomycin**

| Parameter (unit) | Definition | Estimate (RSE%) | Shrinkage |
| --- | --- | --- | --- |
| Q (L/hr) | Distribution flow | 5.36 (8%) |  |
| CL (L/hr) | Systematic clearance | 6.72 (2%) |  |
| V1 (L) | Central Volume of Distribution | 12.7 (4%) |  |
| V2 (L) | Peripheral Volume of Distribution | 8.69 (4%) |  |
| K_13_ (hr^-1^) | ELF uptake rate constant | 0.0032 (21%) |  |
| K_31_ (hr^-1^) | ELF efflux rate constant | 4.04 (22%) |  |
| K_34_ (hr^-1^) | AM uptake rate constant | 0.0062 (16%) |  |
| K_43_ (hr^-1^) | AM efflux rate constant | 0.321 (15%) |  |
| IIV- CL (%) | Interindividual variability on Cl | 12.04% (25%) | 4% |
| IIV- V1 (%) | Interindividual variability on V1 | 16% (31%) | 13% |
| σ^2^ (proportional) | Residual variability | 0.0311 (7%) | 5% |


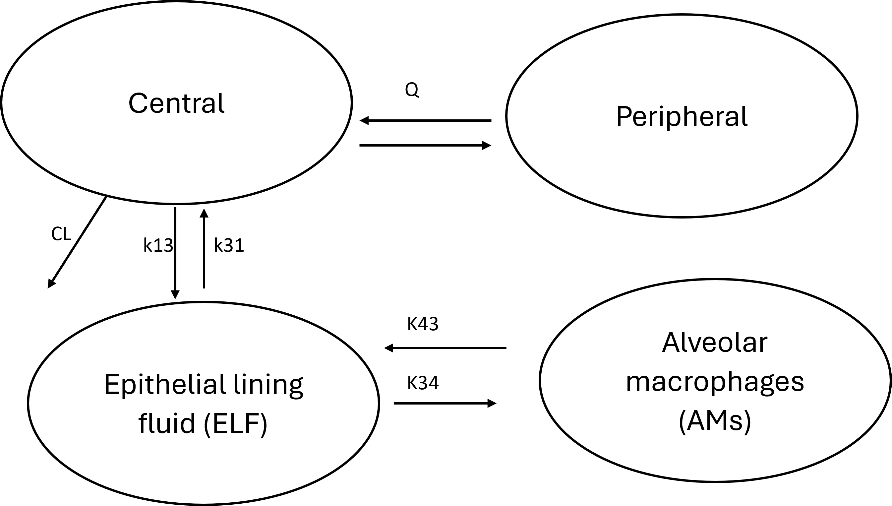


**Figure S1:** Model structure of fosfomycin using plasma, ELF, and AMs samples. Q represents the distribution flow between the central and peripheral compartment, k_13_ and K_31_ represents the ELF uptake and efflux rate constant, respectively, while the K_34_ and K_43_ represents the AM uptake and efflux rate constant, respectively. The volume of the ELF and AMs were fixed to their reported physiological values.

Plasma

ELF

AMs


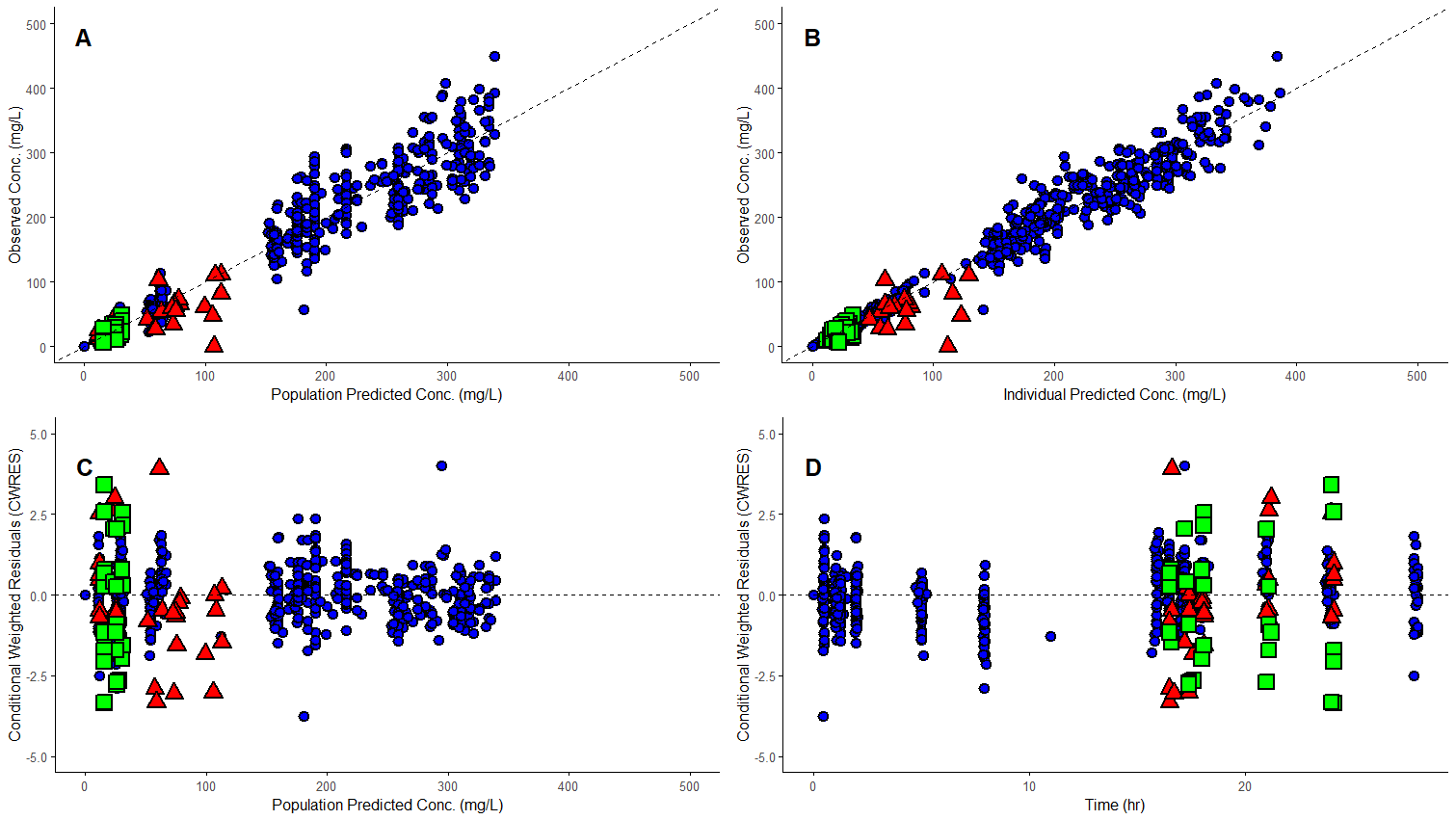


**Figure S2:** Goodness-of-fit plots for the final four compartmental population PK model of fosfomycin. (A) Observed versus population-predicted fosfomycin concentrations, (B) observed versus individual-predicted fosfomycin concentrations, (C) conditional weighted residuals versus time, (D) conditional weighted residuals versus population-predicted fosfomycin concentrations. Dashed black lines represent lines of identity in (A) and (B), or zero line in (C) and (D).

**Figure S3:** The chemical structure of the phosphatidylcholine, the main surfactant in the ELF space. The structure was drawn using ChemDraw (version 22.0.0.22).

**Table S3: Heatmap of PTA of fosfomycin at different MIC values following different dose regimens in healthy and pneumonia subjects, with the two different PK/PD targets associated with the main pathogens that cause HAP. (green, PTA ≥90%; yellow, PTA 80-89%; orange, PTA 79->50; red, PTA ≤50%)**
